# “It’s like ‘liquid handcuffs”: The effects of take-home dosing policies on Methadone Maintenance Treatment (MMT) patients’ lives

**DOI:** 10.1186/s12954-021-00535-y

**Published:** 2021-08-14

**Authors:** David Frank, Pedro Mateu-Gelabert, David C. Perlman, Suzan M. Walters, Laura Curran, Honoria Guarino

**Affiliations:** 1 Behavioral Science Training in Drug Abuse Research, NYU Rory Meyers College of Nursing, 380 2nd Avenue, Suite 306, New York City, NY 10010 USA; 2 Center for Drug Use and HIV/HCV Research, NYU School of Global Public Health, New York City, NY USA; 3 Graduate School of Public Health and Health Policy, City University of New York, New York City, NY USA; 4 Icahn School of Medicine at Mount Sinai, New York, NY USA; 5 School of Global Public Health, New York University, New York City, NY USA

**Keywords:** Methadone Maintenance Treatment (MMT), Take-home doses, Methadone clinics, Harm reduction, Patients’ rights, Stigma

## Abstract

**Background:**

Methadone Maintenance Treatment (MMT) is widely recognized as one of the most effective ways of reducing risk of overdose, arrest, and transmission of blood-borne viruses like HIV and HCV among people that use opioids. Yet, MMT’s use of restrictive take-home dose policies that force most patients to attend their clinic on a daily, or near-daily, basis may be unpopular with many patients and lead to low rates of treatment uptake and retention. In response, this article examines how clinics’ take-home dosing policies have affected patients’ experiences of treatment and lives in general.

**Methods:**

This article is based on semi-structured, qualitative interviews with a variety of stakeholders in MMT. Interviews explored: reasons for engaging with, or not engaging with MMT; how MMT is conceptualized by patients and treatment providers (e.g., as harm reduction or route to abstinence and/or recovery); experiences with MMT; perception of barriers to MMT (e.g., organizational/regulatory, social) and how MMT might be improved to support peoples’ substance use treatment needs and goals.

**Results:**

Nearly all of the patients with past or present MMT use were highly critical of the limited access to take-home doses and consequent need for daily or near daily clinic attendance. Participants described how the use of restrictive take-home dose policies negatively impacted their ability to meet day-to-day responsibilities and also cited the need for daily attendance as a reason for quitting or avoiding OAT. Responses also demonstrate how such policies contribute to an environment of cruelty and stigma within many clinics that exposes this already-stigmatized population to additional trauma.

**Conclusions:**

Take-home dose policies in MMT are not working for a substantial number of patients and are reasonably seen by participants as degrading and dehumanizing. Revision of MMT regulations and policies regarding take home doses are essential to improve patient satisfaction and the quality and effectiveness of MMT as a key evidence-based treatment and harm reduction strategy.

## Introduction

Methadone Maintenance Treatment (MMT) is widely recognized as one of the most effective ways of reducing risk of overdose, arrest, and transmission of blood-borne viruses like HIV and HCV among people that use opioids [1–4]. Yet it is unpopular among many people who use illegal opioids [5, 6] and its effectiveness has been hindered by consistently low rates of uptake and retention [7] that prevent it from meeting its potential as a harm reduction and public health intervention [8, 9].

Research suggests that clinic policies that provide limited access to take-home doses, and the corresponding need for patients to attend their clinic on a daily or near-daily basis, is a primary driver of patient dissatisfaction [10, 11]. Drug user rights groups, such as the Urban Survivors Union, have also argued that the lack of access to take-home doses is unethical and cruel, and that it is only because of the powerful stigma against people who use opioids that such policies are considered acceptable [5]. Moreover, there is a strong feeling among members of such organizations, that people who are not on MMT themselves, or directly connected with someone who is, simply do not understand how disruptive and counter-productive such policies are to the lives of patients [5]. Yet, there are very little data describing the effects of take-home policies on patients’ lives, including their experiences with treatment.

Thus, it is particularly important to investigate this issue in a way that centers the voices and experiences of people who use drugs (PWUD) and people on MMT especially in light of the long history of ignoring the views of PWUD in the design of substance use treatment programs and policies [5, 12, 13].

To address this gap, this study used semi-structured qualitative interviews with PWUD, most of whom were either currently or previously in MMT, and with treatment providers, to examine how clinics’ take-home policies have affected patients’ experiences of treatment and lives in general. Findings suggest that the current approach that requires frequent visits to MMT clinics is impractical and counter-productive, and, as such, serves as a barrier to treatment uptake and retention. In conclusion we suggest some potential policy changes for addressing the problem.

## Background

### Regulations

Federal regulations mandate that when patients begin MMT they must go to their clinic every day (or six days per week if the clinic is closed on Sundays) to ingest their daily methadone dosage under direct observation by clinic staff [14]. As patients accumulate time at the clinic and test negative for illicit opioids on screens given at least once a month they are provided “take-home doses” of methadone to be used on days when clinic attendance is not required. Although federal regulations stipulate how often and under what circumstances take-home doses can be provided, individual clinics can adopt stricter policies if they choose [14]. For example, while patients can typically earn a maximum of 14 days’ worth of take-home doses (except for the few clinics that offer “medical maintenance” which provide a maximum of 28 take-home doses), many clinics choose to offer a maximum of only one week’s supply or less and require long periods of attendance and negative drug tests results to qualify [11].

Similarly, while the Substance Abuse and Mental Health Services Administration (SAMHSA) provides treatment providers with recommendations on how to determine patient eligibility for take-home doses, clinics have wide latitude to determine which patients meet that standard. For example, some clinics will not provide any take-home doses to patients who use cannabis or drink alcohol [15]. Thus, there is substantial diversity among individual clinics in regard to how many take-home doses are allowed and under what circumstances they are provided [11, 16].

Although there are no national data on how many patients receive take-home doses, studies have found that most patients receive very few [11, 17]. According to a recent study on take-home dose provision, while the percent of participants receiving any take-home doses (pre-Covid-19) ranged from 56 to 82%, the majority of that group (59.8%) were only receiving 1–2 days, meaning they were required to attend their clinic for the remaining 5–6 days [11].

Studies have shown that the absence of take-home doses can be a barrier to initiating treatment and that patients are more likely to discontinue MMT when take-home doses are removed [18, 19]. There is also no evidence that requiring daily or near-daily attendance improves patient outcomes [20].

### The effects of Covid-19 on take-home doses

The emergence of the Covid-19 pandemic and associated social distancing mandates placed a newfound focus on the provision of take-home doses because of the often-crowded physical spaces at most MMT clinics. In response, SAMHSA instituted amended guidelines allowing clinics to provide a greater number of take-home doses to a greater number of patients, thereby reducing the need for daily attendance [21]. The new policy granted clinics blanket authority to provide all “stable” patients with up to 28 days of take-home medication. Patients seen as less stable, but still capable of safeguarding and handling their doses, were eligible for up to 14 days of take-home doses [21].

Recent research indicates that the increased access to take-homes was highly beneficial to most patients and that there was little evidence of diversion [11, 22]. However, the new policies were adopted inconsistently and, in some cases, existing policies were not modified at all [23]. Nevertheless, drug user advocates see the expanded access as an opportunity for change, and have pushed to make the new standards permanent [24, 25].

## Methods

### Participant recruitment and interviews

This article is based on semi-structured, qualitative interviews with members of the following groups: a) people currently on MMT (*n* = 13); b) people formerly on MMT (*n* = 4); c) people that use illegal opioids who have never been in Opioid Agonist Treatment (OAT) (*n* = 4); d) MMT treatment providers (*n* = 5); e) buprenorphine treatment providers (*n* = 5); and f) people who work in government agencies that regulate MAT (*n* = 5).

Participants were recruited using a combination of purposive and snowball sampling, between June and October, of 2020 in New York City [26, 27]. Recruitment was conducted using a variety of strategies including: an advertisement placed on craigslist; flyers at clinics or harm reduction organizations; and through word-of-mouth.

The study sample was diverse in terms in age, race, socio-economic status and gender. However, since participants were sampled from the New York City area, participants experience of treatment was primarily in an urban setting. As such, most had access to MMT providers, available public transportation, and comparatively short commuting distances to clinics.

Interviews lasted between 60 and 90 min and were conducted by video. Interviews were audio recorded (and video recorded if the participant consented to this) and transcribed later. Interviews explored the following domains: participants’ substance use history; reasons for engaging with, or not engaging with, and retention within MMT; how MMT is conceptualized by patients and treatment providers (e.g., as harm reduction or as a route to abstinence and/or recovery); how MMT is practiced; experiences with MMT; perception of barriers to MMT (e.g., organizational/regulatory, social) and how MMT might be improved to better support individuals’ various substance use treatment needs and goals.

All participants’ names included in this article are pseudonyms and all participants provided informed consent to participate in this study.

### Data analysis

Data was coded and analyzed by Dr. Frank using AtlasTi, version 8. Analysis was guided by a thematic approach that aimed to organize data into meaningful categories based on the aims of the study and existing literature and informed by the first author’s lived experience using illegal opioids and in MMT [28].

Dr. Frank has been on MMT for approximately 17 years and received services at two clinics: one in Chicago, IL and one in The Bronx, NY. Moreover, as he has done in previous studies, Frank regularly disclosed his status as someone with lived experience of opioid use who was currently on MMT to study participants [9, 15]. Although he has discussed many of the methodological issues associated with this choice in other articles (see for example [29], in short, the authors believe that by disclosing Frank’s shared history, he was able to develop a level of comfort and trust with study participants that facilitated more robust and honest conversations and a richness of data that would not have been possible otherwise. There is an extensive literature describing the mistrust that PWUD often feel towards public health and substance researchers that similarly recognizes the importance of research conducted by and involving community insiders [30–32].

### Theoretical approach

Consistent with Dr. Frank’s lived experience with opioid use and MMT, the authors used a situated theoretical approach to data collection and analysis. Situated approaches are those that acknowledge the positionality and power relationships existing between researcher, subject, and participant [33–35]. They are often used when studying groups that are structurally and/or ideologically marginalized, and generally place a greater emphasis on transparency and reflexivity than on neutrality and objectivity. Situated approaches are also more comfortable with the political and activist concerns of research than other methodological approaches that focus primarily on uncovering objective knowledge. In situated approaches, challenging power is seen as a valuable part of the process [33, 36].

## Results

### Patients’ experience of restricted access to take-home doses

Nearly all of the patients with past or present MMT use were highly critical of the limited access to take-home doses and consequent need for daily or near daily clinic attendance. It was by far the most commonly reported complaint from patients and former-patients. Participants often compared the restrictions to jail and/or used the pejorative that MMT was like “liquid handcuffs”. Participants’ responses also demonstrated significant anger, resentment, and a sense that their experiences were not understood by the “outside world”. For example, patients said:

I hate it [MMT] because it’s like liquid handcuffs. Say you want to go somewhere for a few days, you need take-homes and if they won’t give them to you, there’s nothing you can do. The outside world, or people that are not on MMT don’t really understand what that’s like…. Take-homes are the biggest thing [problem], everyone has trouble with them, whether it’s losing their job, or they can’t go out of town, or they’re just late, or sick. And then especially, with hurricane Sandy. Whenever there’s going to be a storm in the forecast, I specifically hide some of my pills so that I’ll have something, just in case.

Samantha, female, previously on MMT

I need, I want to get off it [MMT]. It’s like being in jail. Because you have to go every goddam day.

Lisa, female, currently on MMT

The only time – yeah, the only time you got a take home was something like Christmas Day and New Year’s Day. And, yeah, and other than that, you went and there was only one program and you could be three hours away on the other side of the island and it didn’t matter.

Genene, female, currently on MMT

Having to be at the clinic everyday made it particularly difficult for patients to maintain steady employment, attend school, or manage their daily lives. Since most clinics have limited hours of operation, often early in the morning, it regularly impacted their ability to get to work or attend to other daily responsibilities on time. Moreover, since many participants hid their participation in MMT from employers, they often had to lie on occasions when they were late to work due to clinic attendance. That clinic policies had such a negative impact on participants’ ability to work led to strong feelings of injustice among patients and a sense that they were not being treated in a fair or decent way. For example, participants described this issue in the following ways:

So basically, the idea is to get back to living your regular life, you know? But, in a way, it kind of deters you from doing so sometimes, you know? And you know, and especially at first, I had to go even on Saturdays. I mean, how the hell would I do a regular job like that, you know? How could I get a nine to five, right? You know, I don't know that I could definitely be on time. I've seen it happen. Like, my brother for instance, I've been lucky, kinda, so far, that I've been able to try to make it work, you know?

Dean, male, currently on MMT

Because when they tell you – not only that you have to go there every day, they take control of your life. You can't go on vacation, you gotta tell them when you're going, and you gotta ask permission**.** You know, and then you gotta explain to people why you're always late. I need to – they don't look at you good when you tell them you're on methadone program, but I have to go every day.

Nina, female, previously on MMT

Like say, I have to be at work at 7 o'clock. [The] place opens at 6:30, and then by the time I get out of there, it's already 7 o'clock and I'm supposed to be at work at seven, you know.

John, male currently on MMT

Thus, patients characterized the need for daily or near-daily clinic attendance as antithetical to their efforts to adopt a more stable, and in particular employed, life and to their ability to effectively manage their day-to-day responsibilities. It also adversely impacted their quality of life not only through the daily labor of clinic commutes but also through the stress and anxiety that such policies created in their lives.

### Impact of MMT’s organizational structure on patients’ ability to plan for life activities

Difficulties with daily attendance were exacerbated by the often inconsistent and hard-to-plan-for organizational structure of most clinics. Patients reported that long lines, a lack of communication, and on-the-spot changes in patient requirements, meant that people could not effectively anticipate how long each visit might last. For example, patients were often informed of mandatory meetings with their counselor upon arriving at the clinic. They were rarely given information on the length of time they would have to wait or on how long their appointment was expected to last. They were also unable to postpone or re-schedule such appointments since most clinics prevented patients from dosing in such circumstances. Moreover, patients knew that leaving would likely result in disciplinary actions and would almost certainly mean the loss of any currently held take-home doses. For example, Dean, who is currently on MMT described the effects of such practices in the following way:

I've had numerous times, like, when I was going, three times a week or whatever, [and] I would have a counselor session, so now, not only am I standing on the line, and you know, getting medicated, which is gonna take 15 to 20 minutes, now I gotta stop and see the counselor, and they could be with somebody else. I mean, I've had instances when I went in and out, but I've also had instances when I go – walk in there thinking I'm gonna be out in 15 minutes, and I've been in there for freaking an hour and a half, you know? I mean, that's a big chunk of your day. How am I supposed to – you know, how am I supposed to hold down a normal job, and you know, deal with that when it's not even scheduled? You know what I'm saying?

Dean, male, currently on MMT

Patients were also randomly required to make additional clinic visits, known as “callbacks” in order to prove that they had to correct amount of medication and thus, were not selling it or using it too fast. As Kathy, a 65-year-old woman on MMT described:

What they did was the call backs were five days a week you had to call a phone number at 6:00 PM. And if it was your number, the next morning you had to go there with your bottles. And it was like, you know, I was an executive for Fortune 5 Company. I work for [a well-known company] and I might find out the night before I had to be in the New York office for board meeting. And I was like, “How can I live like this?”

Kathy, female, currently on MMT

Similarly, participants reported that clinics were often unwilling to provide take-home doses for life events, such as weddings, funerals, or medical emergencies. Moreover, since clinics often employed a complex and time-consuming committee structure to make such decisions, patients were unable to plan for such occurrences and many described having missed out on important family gatherings and events. For example, when asked if their clinic would provide take-home doses for an emergency, Todd said:

I don’t think so [that my clinic would give me take-homes for a family emergency], no. Because the thing is, it would have to – my counsellor would have to have a sit down with the committee, they would have to talk about it, so if it's an emergency situation, and I have to go, that timeframe is not – you know, I don’t have time for that. So, if I call them now, and I'm like, "I got a flight to Florida, I got to go in, like, a half hour," you know, I'm not going to get the take-home doses.” If something were to happen where, like, you know, my father is getting up there in age, so if, God forbid, something was to happen, and I had to [go see him], my only option is to figure out how, you know. I'm not going to get sick when I go to Florida, you know what I mean? And I think it's going to be – I don’t think I'm going to have any luck talking to them [the clinic], so the only option I'm left with is, you know, figuring out on my own devices [obtaining opioids illegally], you know what I mean?

Todd, male, currently on MMT

These difficulties are often exacerbated by the expense, complexity, and limited availability of clinics that provide guest dosing to out-of-town patients as well as by the need for many patients to keep their participation in MMT a secret from family members.

As participants’ responses demonstrate, patients experience significant frustration over polices which they saw either as completely incomprehensible or as an expression of stigma and dislike of PWUD. Many expressed the view that the take-home structure made improving one’s situation while on MMT a nearly impossible task.

Moreover, as Todd described, such practices put patients in the position of having to choose between missing out on important life events, trying to attend them while in withdrawal, or obtaining opioids on the illicit market, and thus risking a future failed clinic drug screen which would then result in the removal of any take-home doses that patient was currently receiving (as well as the other risks associated with buying and using illegal opioids).

In some cases, clinicians’ treatment of patients was even cruel and dehumanizing. For example, Nina, who is no longer on MMT, described a harrowing and degrading experience in which she was made to wait in line after her water broke during pregnancy.

The last clinic, I was at, I was pregnant, and I had my water break in line, and it was a long line. I asked them to put me to the front, and they made me wait in line. I told them my water broke and they made me stay in line. Instead of medicating me, they made me stay in line with everybody.

Nina, female, previously on MMT

### Lack of take-home doses creates a deterrent to participation in MMT

People who had quit MMT, or who have never been on it, often described the need for daily attendance as their primary reason for avoiding or quitting treatment. They pointed out that in combination with long commuting times to the clinic, that everyday attendance is unrealistic as a workable, long-term solution. For example, one participant who said that he “would have jumped on treatment in a second” described why he chose not to in the following way:

You have to go to go there every single day and get doses, which is a complete, that’s a nightmare. That’s going to probably be the number one reason people don’t want to get on Methadone. You got to go, you have to go stand there in the morning, wait to get dosed and it just sounds like a real pain. I’d much rather see a doctor once a month, get a prescription and deal with it like that… I lived in Pennsylvania, and at the worst time, I would have jumped, I would have jumped on treatment in a second. But the closest clinic was in Lancaster and that meant that I would have to drive up there every day, which it was a little over two hours each way so, I would have to do that every day.

Edward, male, never been on MMT

Others, echoing Todd’s comment that restrictive take-home policies encourage patients to continue using illegal opioids, pointed out that restrictions on MMT made it so onerous that it was easier to simply obtain opioids illegally. For example, Nina, a 63-year-old woman who had previously been on MMT explained:

That was one of the things that drove me away, that I would rather be on heroin than be on methadone. Cuz I wanted to be free to do what I want to do when I want to do it, and I – and could take them with me, and go where I want… [When I was on MMT] I was like a double slave. Like, you're a slave to the heroin already. And you're on methadone, you're a slave to the methadone and the clinic.

Nina, female, previously on MMT

### A financial incentive for not providing take-home doses

Patients, treatment providers and individuals from government agencies that regulate MMT all described a variety of ways that clinics were financially disincentivized from providing patients with take-home doses. For example, patients described how insurance, and the different ways that companies reimbursed clinics, affected how often they were required to come in. Patients also regularly described clinics as “businesses” and saw treatment decisions as being motivated primarily by financial rather than healthcare-related concerns. They reported the following:

This was kinda shitty. I remember I earned up to two weeks [of take-home doses], and I was self-paying, you know? And then what happened was I started working a nine to five again, and I qualified for – not Medicaid, but the, like, family health plus or something like that. So, because they [Dean’s new insurance company] paid less [than he previously paid as an out-pocket customer], they [the clinic] wanted me to now come once a week. Even though I was still just as abstinent, they wouldn't get paid as much since I wasn't self-paying anymore. So, they wanted to bill every week. And my counselor basically told me the truth. She's like, "Look, they're not gonna ok this." And I thought that was kinda fucked up, you know? You know, it's like, "Really?" You know? It's like – you know, cuz – I mean I understand that [it’s a] business, absolutely, but you know.

Dean, male, currently on MMT

I go once every two weeks now that's only, because of the COVID and, because of the fact that I'm on Medicaid. Medicaid—if I was on some other type of insurance, I would only have to go once a month regardless of the COVID but, because Medicaid will not pay for you to come—they won't pay the clinic the money unless you show up.

Allison, female, currently on MMT

But it also is a business, and so, sometimes, when you go to a counsellor, or you go to somebody from the program, I feel you kind of get a little sided – not honest – because they don’t want you to get off, they want you to keep coming, you know what I mean? It's a business before anything else.

Todd, male, currently on MMT

Treatment providers and respondents who work in government organizations that administer MMT also described a structure of financial incentives linked to clinic attendance. While indicating that the logistics of clinic billing are complex and subject to differences in state and local regulations and between individual clinic policies and practices, they nevertheless described a reimbursement structure that disincentivized the provision of take-home doses. For example, they reported the following:

I'm wondering if you've looked into this – that the amount of times, the number of times people go is tied into the reimbursement that clinics get, and that alone is a big deal. I really can’t claim to understand, it is the whole billing process. [But] I think the way that programs are able to bill generally is badly set up for people who are stable and receiving 28 days delivery, but it’s something to do with paying for, you can get the money for the medication and then you get money for the counseling or the other kind of support services, that clinics have been reluctant to either increase people’s take-home doses because of a financial barrier.

Dale, female, government employee

I would say that they are not supported properly with the proper financial reimbursement that supports a proper successful model… So, it's not necessarily that they're incentivized to bring people in every day. It's that they're not reimbursed with a structure that allows them, that supports them financially, to give out 28 days to successful patients. So just to give you a quick idea, you get paid -- let's say it's $20 every time someone comes just for the medicine, for a medication visit, but the first visit of the week is $40, let's say. So, if you came once for 28 days and that's all you got, you would get $40 for that visit.

Sofia, female, treatment provider and advocate

It seems to us even in the way that OASAS [Office of Addiction Services and Supports] and other folks were describing it was the system basically is not set up well for people who are stable, because the reimbursement is less. And so, there is less incentive for any of these clinics, which are all money-making entities, to extend peoples’ take-home doses.

Christine, female, government employee

Thus, despite the many variables that likely play a role in this issue, participants were confident that clinics’ take-home dose policies were directly related to an institutional reimbursement structure that incentivizes clinic attendance to generate MMT program revenue and thus disincentivizes the provision of take-home doses.

### Providers’ views on take-home doses

Many treatment providers, particularly those who worked primarily with buprenorphine, agreed with patients that the current approach is impractical and poorly suited to addressing patients’ needs. For example, they stated that:

[You shouldn’t] have to go every day for however indefinite number of years to a place that's 90 miles away from your house, and you've got to ride public transportation or whatever it is. Yeah, no. Our closest methadone clinic from my office is about a half hour. From the central place for most of my patients based on where they live in the zip code. It's a two-and-a-half-hour round trip to go to a methadone clinic so you can't work, you can't have a job. You can't go to school. You get your methadone and go home. You do that every day. Yeah, the system is broken.

Richard, MD, male buprenorphine provider

Well, the need to be at the clinic up to six times a week is really not conducive to living a life that’s away from an environment which is going to attract drugs. There’s a certain social control component that goes into that, and even though clients might be encouraged to remain abstinent certainly they’re going to pass their fair length of drug dealers when they go to look forward to clinic. And make acquaintances of people who are at the high possibility of being active users, and also even if you want to work how do you work when you have to go to your clinic five days a week and medical six days a week. [We need more] flexible scheduling.

Anthony, MD, male MMT provider

The views of clinicians provide an important point of triangulation that gives added credence to the testimony of patients, particularly since they align so closely with patients’ responses.

## Discussion

These findings provide an in-depth examination of the many ways that restrictive take-home dose policies negatively impact the lives of people on MMT. They describe a situation whereby the organizational approach to take-home doses almost guarantees that patients will encounter significant life disruptions that result in impediments to their ability to maintain employment, travel and respond to important life events.

Very few people in any walk of life would be able to show up to a non-work appointment at 6am every single morning and even less so when administered in the rigid, top-down and punitive manner described by patients. Indeed, it is only because the first author was able to find one of the few clinics that does offer 28 days of take-home doses, that he was able to attend graduate school and pursue a professional career. Had Dr. Frank been required to attend clinic every day, or even a few times a week, for years on end, graduate school and a career would have been entirely impossible.

By revealing how poorly people on MMT are often treated, the study findings also reveal the extent that they are marginalized. Patients’ descriptions evoke Giorgio Agamben’s socio-philosophical work on “bare life”, which highlights that certain (marginalized or oppressed) populations are seen by others as being outside of, or a “state of exception” to, the boundaries of human citizenry and thus not deserving of basic human rights [37, 38]. Agamben developed this set of concepts to help explain how certain groups, such as prisoners in Guantanamo Bay, are treated with little regard to standards of decency. Descriptions by MMT patients of being forced to stand in line after clearly going into labor or being denied the opportunity to attend a family members’ funeral—particularly when done in the context of the biological power that clinicians wield over patients through their dependence on methadone [39, 40]—show a particular cruelty and dismissiveness of this populations’ human rights, as though they are undeserving of the same standards of ethical humane consideration and respect that are generally and appropriately expected in patient-provider, and indeed -most, relationships.

Similarly, the literature on medicalization describes how medical personnel derive power through the socio-cultural framing of behaviors, particularly those activities seen as deviant, like substance use, as medical problems [41, 42]. Not surprisingly, treatment providers often rely on addiction-as-disease narratives that position PWUD as inherently disordered and in need of the clinics’ firm hand, to justify such polies. Yet, Frank’s previous work has contended that this is a mischaracterization of the reasons that PWUD use and benefit from MMT. Rather he has argued that MMT is better understood as a survival strategy that PWUD use in a variety of ways to maintain their use of opioids and reduce the harms of criminalization of drug use [9, 43]. In other words, whether patients pursue abstinence or not, MMT provides them with a shelter from harms stemming from the criminalization of drug use. Similarly, Frank & Walters have problematized the notion of consent in MMT by pointing out the ways that structural, legal, and cultural forces constrain the decisions of people who use illegal opioids [29]. In this light, restrictive take-home policies, and those who enforce them appear as yet another structural barrier preventing PWUD from obtaining safe access to opioids.

The solutions to this problem are, in many ways, not complicated. Programs must be made tolerable to patients, otherwise they will not use them. This is borne out by the consistently low rates of use and retention and correspondingly high rates of patient dropout in MMT [44]. However, since restrictions are tied to an institutional structure that disincentivizes take-home dose provision, part of the solution requires creating an institutional reimbursement system or health care delivery model that supports, and at a minimum does not structurally disincentive, increased take-home doses. Advocates from the Urban Survivors Union, a harm reduction and drug-user rights organization recently published a “Methadone Manifesto” that also recommends abandoning requirements that limit take-home dose provision to those with long records of attendance and negative drug tests, and similarly, that peoples’ take-home doses not be suspended or rescinded for positive drug screens [5].

In addition, changes should be made that allow for the provision of methadone in a manner similar to buprenorphine, another maintenance-based medication for people that use opioids. Because they are regulated differently, buprenorphine providers have far more latitude to determine the most appropriate take-home schedule for their patients and generally provide take home doses more often than MMT clinics [45]. In fact, research shows that some PWUD choose buprenorphine over MMT, despite a preference for methadone, because of the access it provides to take-home doses, and that—since buprenorphine is only a partial agonist and methadone is a full-agonist—such choices could lead to negative healthcare outcomes [29].

Opponents to providing increased access to take-home doses most often cite fears of diversion [46]. However, research suggests that this consideration is both exaggerated and mischaracterizes the complex risk environment that MMT addresses [47–49]. First, studies show that diversion is uncommon and happens more often among people using opioids for pain than for substance use [48, 50]. Recent studies examining expanded access to take-home doses during Covid-19 also found that diversion was rare [11, 22]. Second, concerns over diversion ignore the more significant risks of *not* providing take-home doses. As our findings demonstrate, the lack of take-home dose provision incentivizes patients to obtain opioids illegally where they will be exposed to the much greater risks associated with illegal and unregulated opioid use [51–53]. Restrictive take-home policies also encourage treatment discontinuation and discourage PWUD from engaging in MMT. In short, the risks of diversion, even when it happens, are far less than those produced by the current approach to MMT.

Further, the common clinic policies of using random drug testing or pill/bottle count spot checks and ‘call backs’ to assess if patients are potentially using illicit substances between visits, or potentially diverting methadone, should be reconsidered. While current US Federal guidelines require random urine checks as part of “diversion control plans [14], quantitative data to ascertain the degrees to which these practices either actually improve relevant outcomes or contribute to non-retention in treatment are scarce [54]. Our data demonstrate the extent to which these practices function as ‘part of the handcuffs’ and serve as barriers to job attendance and other social responsibilities. Further data to assess the holistic outcomes of random callbacks and spot checks are needed to soundly inform the design of truly evidence based, effective and humane, clinic policies.

Individual clinics can also make changes that would help to improve the burden on patients. For example, using a better organizational approach to scheduling patient appointments would improve many of the difficulties patients face. Similarly, clinicians can be more respectful of patients’ time commitments and outside-of-the-clinic responsibilities, and avoid using punishment, or the threat of punishment, as a response to scheduling issues.

Finally, there is a need for more detailed, publicly-available information on MMT clinics’ take-home dose policies and practices. SAMHSA should collect clinic-level data on how many patients receive take-home doses and according to what schedules. Similarly, clinic level data on individual clinics’ take-home policies, such as the maximum amount of take-home available and what kinds of metrics are used to ascertain whether a patient qualifies for addition take-homes, should also be made more readily available. This would help researchers and policymakers to develop a clearer picture of take-home provision and better determine which policies are working and which are not.

This paper has relevant limitations. It is based on a relatively small sample size. Similarly, and as with all qualitative research, results can not necessarily be generalized to the larger population of people on MMT. Additionally, as noted in the Methods section, it utilized an insider’s perspective which informed the data collection and analysis. Lastly, we would have liked to include demographic information about participants’ race and age in the interview quotations and explored its role in this issue. However, partly because of data collection difficulties that arose as a result of COVID-19, we were unable to collect this information for all of the participants and thus, decided to not include it for any of the quotations.

In conclusion, these data clearly demonstrate that take-home dose policies in MMT are not working for a substantial number of patients, are reasonably seen by participants as degrading and dehumanizing, and directly contribute to both non-engagement in MMT, MMT non-retention, and to significant difficulties with patients’ employment and in their lives. MMT’s many benefits are well known and supported by decades of evidence, and recognized by many PWUD (55). Yet, if MMT is made so onerous, degrading, and difficult that patients conclude that they cannot remain, or that it is not worth remaining, in treatment and live a ‘regular’ stable, fulfilling life, then they will not. Rather, and has been seen for decades, they will continue to simply ‘remove their liquid handcuffs’ and disengage from MMT, and return to obtaining opioids solely through the riskier illegal market. Revision of MMT regulations and policies regarding take home doses, drug testing and pill/bottle counts, and general operations are essential to improve patient satisfaction and the quality and effectiveness of MMT as a key evidence-based treatment and harm reduction.

## Data Availability

The datasets generated during and/or analyzed during the current study are not publicly available due to privacy concerns but are available (in a de-identified format) from the corresponding author on reasonable request.
